# N-Propargyl caffeate amide (PACA) prevents cardiac fibrosis in experimental myocardial infarction by promoting pro-resolving macrophage polarization

**DOI:** 10.18632/aging.102959

**Published:** 2020-03-23

**Authors:** Yuanyuan Cheng, Dan Luo, Yingke Zhao, Jianhui Rong

**Affiliations:** 1 School of Chinese Medicine, Li Ka Shing Faculty of Medicine, The University of Hong Kong, Hong Kong, China; 2 The University of Hong Kong Shenzhen Institute of Research and Innovation (HKU-SIRI), Shenzhen, China

**Keywords:** macrophage polarization, cardiac fibrosis, N-propargyl caffeate amide, PPAR-γ pathway

## Abstract

Macrophages control the initiation and resolution of cardiac fibrosis in post-infarction cardiac remodeling. The aim of the present study was to investigate whether N-propargyl caffeate amide (PACA) could suppress myocardial fibrosis via regulating macrophage polarization. By using rat model of isoproterenol-induced myocardial fibrosis, we discovered that PACA could reduce cardiac fibrosis in a dose-dependent manner. To elucidate the anti-fibrotic mechanisms, we examined whether PACA affected pro-inflammatory M1 and pro-resolving macrophage biomarkers in macrophage polarization. As result, PACA reduced the expression of pro-inflammatory M1 biomarkers (e.g., iNOS, TNF-α, CXCL10, IL-6, CCL2 and CD80) while increased the expression of pro-resolving M2a biomarkers (e.g., IL-10, arginase-1, FZZ1, YM-1 and CD163) in LPS-stimulated RAW264.7 macrophages. PACA also suppressed the elevation of M1 biomarker ED1 in the early phase but up-regulated the expression of pro-resolving biomarker ED2 in the later phase. Moreover, PACA reduced the expression of pro-fibrotic TGF-β1 and PDGF-α while maintained or even increased the production of pro-apoptotic MMP-13, MMP-9 and TRAIL. Importantly, mechanistic studies revealed that PACA might promote the switch of macrophage polarization towards a pro-resolving macrophage phenotype via activating PPAR-γ pathway. Taken together, this study suggested that PACA might be a drug candidate for preventing cardiac fibrosis in myocardial infarction.

## INTRODUCTION

Post-infarct cardiac fibrosis is a major risk factor for ventricular arrhythmia, cardiomyopathy, heart failure, hypertension and even sudden death [1, 2]. Fibrosis inhibition may prevent the onset of serious cardiac events, ameliorate ventricular remodeling and improve cardiac functions [3]. Cardiac macrophages are implicated in heart tissue remodeling and repair [4]. Macrophages exhibit remarkable phenotypic and functional plasticity and undergo pro-fibrotic M1 and pro-resolving macrophage polarization after myocardial fibrosis [4, 5]. M1 macrophages produce pro-inflammatory cytokines to exacerbate cardiac injury, promote the proliferation and activation of myofibroblasts, and recruit the circulating fibrocytes [6, 7]. Pro-resolving macrophages not only express TNF-related apoptosis-inducing ligand (TRAIL) and MMP9 to trigger myofibroblast apoptosis [7], but also express fibrolytic proteases including MMP12 and MMP13 to degrade excessive collagenous extracellular matrix (ECM) [8, 9]. It was recently suggested that timely switch of macrophages from pro-fibrotic type to pro-resolving type could improve cardiac repair [10]. Thus, pharmacological approaches are needed for promoting the phenotypic switch of macrophages within the context of myocardial fibrosis.

Macrophage polarization is tightly regulated by cytokines (e.g., IFN-γ, IL-4, IL-13, TNF-α) and exogenous bacterial liposaccharide (LPS) through multiple transcriptional mechanisms including NF-kB, STAT1, STAT6, IRF4, IRF5, PPAR-γ and PPAR-δ [4, 11, 12]. PPAR-γ is a ligand-activated transcription factor to promote anti-inflammatory M2 macrophage polarization in humans and mice [13, 14]. Conditional knockout of PPAR-γ in macrophages or disruption of PPAR-γ in myeloid cells not only diminished M2 macrophage polarization but also initiated the onset of glucose intolerance, insulin resistance and obesity [14]. By contrast, PPAR-γ activation in monocytes increased the population of anti-inflammatory M2 macrophages in atherosclerosis [15, 16]. PPAR-γ agonists preserved the fluidity of the myocardial cell membrane and reduced cardiac injury in hypercholesterolemia rats although pioglitazone could also decrease myocardial infarct size through PPAR-γ-independent mechanisms [17, 18]. These results suggest that PPAR-γ is an important therapeutic target against myocardial infarction.

Caffeic acid is widely present in free or conjugate forms in a variety of fruits and Chinese herbal medicines [19]. Caffeic acid and the related derivatives (e.g., caffeic acid phenethyl ester and caffeic acid methyl ester) exhibit potent antioxidant, anti-cancer, anti-inflammatory and neuroprotective activities [20]. Interestingly, caffeic acid and its derivatives were also documented for cardioprotective potential against myocardial infarction injury [21, 22]. In our previous studies, we initially synthesized N-propargyl caffeate amide (PACA) as a small molecule probe for identifying protein targets, and found that PACA exhibited neuroprotective effect and cardioprotective effect via covalent binding to Keap1 and subsequently activating Nrf2/HO-1 pathway [23, 24]. However, little is known whether PACA could help the resolution of inflammation and the inhibition of cardiac fibrosis via PPAR-γ pathway.

In the present study, we investigated the *in vivo* anti-fibrotic effects of PACA on isoproterenol-induced cardiac fibrosis in rats. We determined whether PACA could inactivate cardiac fibroblasts and reduce cardiac fibrosis. We focused on the effects of PACA on the phenotypic switch of macrophages from pro-inflammatory M1 to pro-resolving macrophage phenotype and the activities of PPAR-γ pathway. The long-term goal was to evaluate whether PACA could serve as a lead drug for the therapy of cardiac fibrosis.

## RESULTS

### PACA attenuated fibrosis of the rat heart

**Figure 1:**
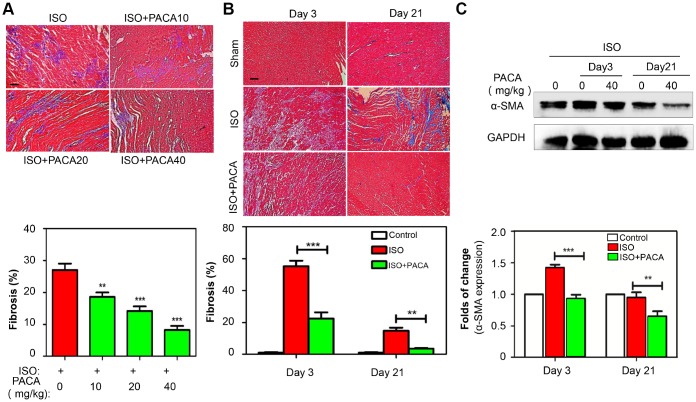
**Anti-fibrotic effects of PACA in a rat model of ISO-induced myocardial infarction.** (**A**) Staining of fibrotic infiltration in the heart tissues. Rats were randomly divided into four groups: ISO and ISO+PACA (10, 20 and 40 mg/kg/day). Following 7-day treatment with PACA, heart tissues were analyzed by Masson’s trichrome staining. Blue stains represent fibrotic infiltration. Original magnification 200 X, scale bar = 50 μm. Cardiac fibrosis was quantified by taking six random images. (**B**) Detection of fibrotic infiltration on Day-3 and Day-21. Following the treatment with PACA, heart tissues were analyzed by Masson’s trichrome staining as described in Panel **A**. (**C**) Determination of α-SMA expression on Day-3 and Day-21. Following the treatment with PACA, heart tissues were analyzed by Western blotting for α-SMA expression (n = 6). Data were expressed as mean ± SEM. *, p<0.05, **, p<0.01, p<0.001 (ISO+PACA vs ISO).

H&E staining was used to examine the pathological changes in the cardiac tissues. As shown in Figure 1A and 1B, almost no broken fibers and inflammatory cells were detected in myocardium from the control group while myocardial cells from the ISO group showed an irregular morphology, broken fibers and infiltrated inflammatory cells. Interestingly, PACA ameliorated the breakage of myocardial fiber and the infiltration of inflammatory cells somewhat in a dose-dependent manner (data not shown). Importantly, PACA at 40 mg/kg significantly suppressed ISO-induced increase in the density of blue-stained collagen fibers between myocardial cells. To determine the effect of PACA on the progression of cardiac fibrosis, we detected the fibrotic biomarker collagen and α-SMA by staining the heart tissues by Masson’s trichrome and monitoring protein levels in a time course. As shown in Figure 1C and 1D, fibrosis in ISO group was readily detectable on day-3 and became more severe on day-7 although fibrosis was resolved to a certain extent on day-21. However, PACA treatment attenuated fibrosis on day-3, day-7 and day-21. On the other hand, ISO increased the protein levels of α-SMA whereas PACA significantly reduced the contents of α-SMA protein on day 3 and day 21 (Figure 1E and 1F).

### PACA regulated the *in vivo* kinetics of macrophage polarization

**Figure 2:**
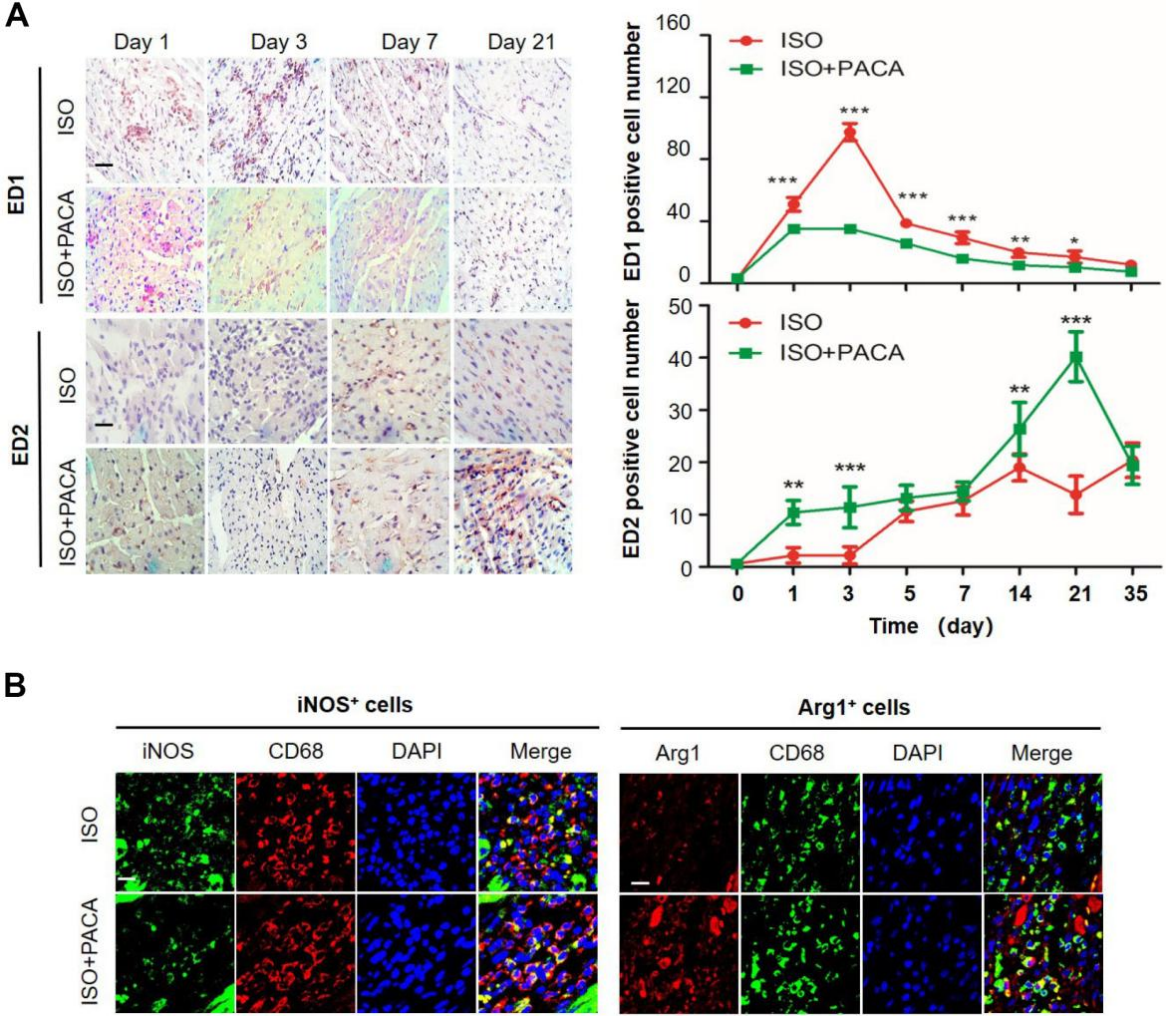
**PACA altered the kinetics of macrophage polarization in hearts.** (**A**) Immunostaining of macrophage biomarkers ED1 and ED2 in heart tissue. The cardiac tissues were probed with antibodies against ED1 and ED2 and detected with ImmunoCruz mouse ABC Staining System. The images were captured under a microscope. Original magnification 400X Scale bar, 50 μm. ED1^+^/ED2^+^ cells was counted from six different images. Data were expressed as mean ± SEM (n = 5). *, p<0.05, **, p<0.01 (ISO+PACA vs ISO). (**B**) Immunostaining of Arg1 and iNOS in macrophages. Following 3-day treatment with PACA, the cardiac tissues were probed with antibodies against CD68, Arg1 and iNOS. DAPI was used to stain the cell nuclei. The tissue sections were imaged under a fluorescence microscope. Scale bar, 20 μm.

To examine the kinetics of macrophage polarization in the hearts, we monitored the expression levels of macrophage biomarkers ED1 and ED2, respectively. As shown in Figure 2A, ISO stimulated the expansion of ED1^+^ cells within and around the affected myocardium. Specifically, the number of ED1^+^ cells was increased from day-1, peaked on day-3. Although rapidly declined after the peak, ED1^+^ cells were still detectable even by day-35. By contrast, As shown in Figure 2A, ED2^+^ cells showed different kinetics from ED1^+^ cells. Although ED2^+^ cells were barely detectable on day-1 and day-3, the number of ED2^+^ cells was increased within the first week after ISO stimulation, peaked on day-14. Interestingly, ED2^+^ cells showed somewhat decrease by day-21 and remained to be greater than the control level by day 35. However, PACA showed differential effects on ED1^+^ cells and ED2^+^ cells. Particularly, PACA reduced ISO-induced accumulation of ED1^+^ cells from day 1 to day 21 while stimulated the presence of ED2^+^ cells from day 1 to day 14 and prolonged the peak time of ED2^+^ cells until day-21. Moreover, the kinetics of ED1^+^ cells and ED2^+^ cells were confirmed by staining M1 biomarker iNOS and pro-resolving M2 biomarker Arg1 in the heart tissues. As shown in Figure 2B, PACA markedly decreased the number of iNOS^+^ cells, while increased the number of Arg1^+^ cells. In addition, we detected the enzyme activity of arginase 1 in the heart tissues of different groups, while PACA barely affected arginase 1 activity in the heart tissues (not shown).

### The *in vitro* effects of PACA on macrophage polarization

**Figure 3:**
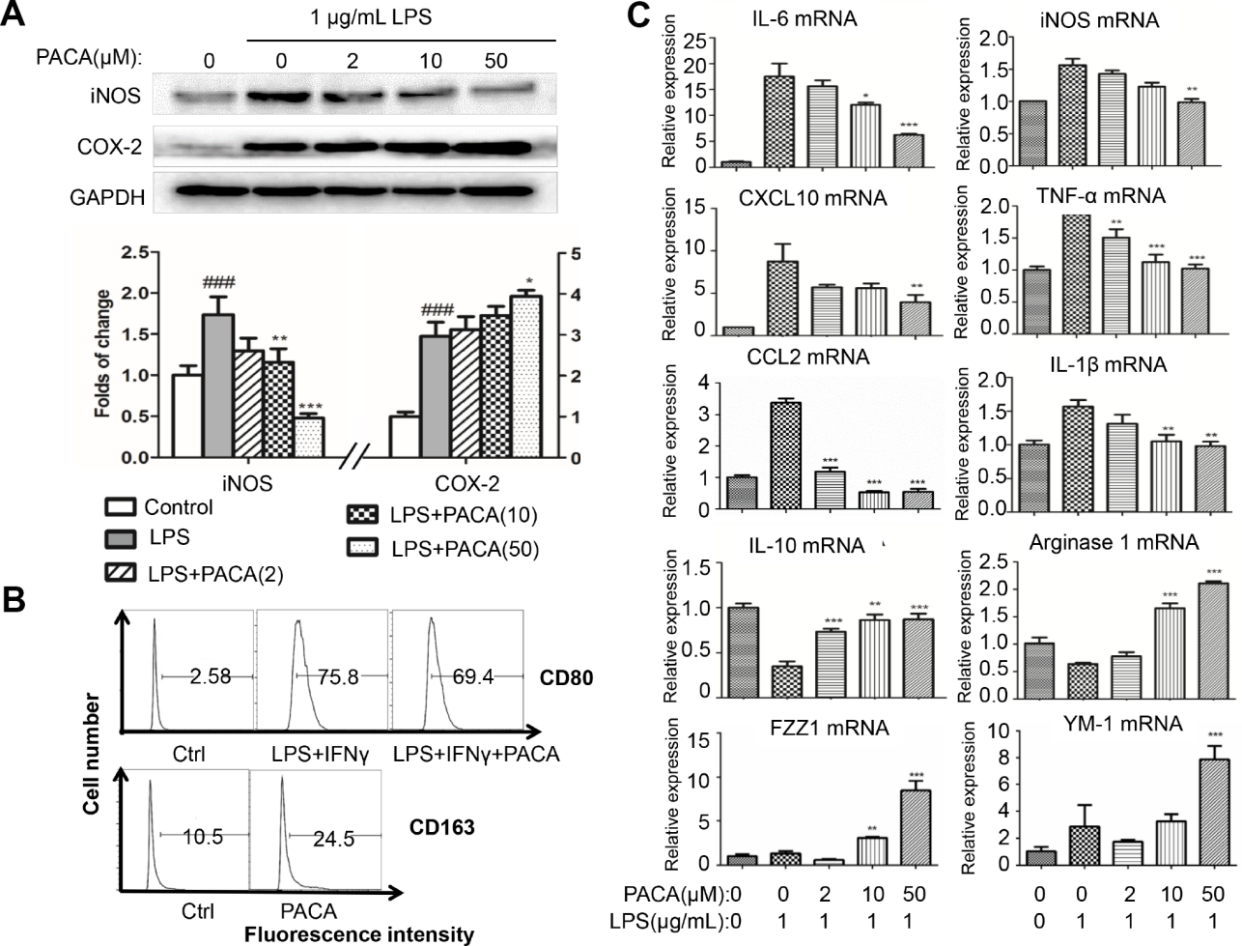
***In vitro* effects of PACA on macrophage polarization.** (**A**) Effects of PACA on iNOS and COX-2 expression in LPS-stimulated macrophages. Following 2 h pretreatment with PACA, RAW264.7 macrophages were co-stimulated with PACA and LPS for another 24 h. The cellular proteins were analyzed by Western blotting for the expression of iNOS and COX-2 (n = 3). **, p<0.01, ***, p<0.001 (PACA+LPS vs LPS alone). (**B**) Flow cytometric analysis of macrophage biomarker CD80 and CD163. Following 2 h pretreatment with PACA, RAW264.7 macrophages were co-stimulated with PACA, LPS and/or IFN-γ for another 24 h. Macrophages were stained with antibodies against CD80 and CD163 and analyzed by flow cytometry. (**C**) qRT-PCR analysis for the mRNA levels of macrophage biomarkers. After the treatment as described in Panel **B**, the total RNAs was isolated and analyzed by qRT-PCR technique. Pro-inflammatory macrophage biomarkers included iNOS, CXCL10, IL-6, CCL2, IL-1β and TNF-α whereas pro-resolving macrophage biomarkers included Arg1, IL-10, FZZ1 and Ym-1 (n = 3). **, p<0.01, ***, p<0.001 (PACA+LPS vs LPS).

To confirm the effects of PACA on macrophage polarization, we monitored the changes of macrophage M1 and pro-resolving M2a biomarkers (Arg1, FZZ1, YM-1 and IL-10) in LPS-stimulated RAW264.7 macrophages. Firstly, as shown in Figure 3A, PACA selectively inhibited LPS-induced expression of iNOS over COX-2. Secondly, we employed flow cytometry to verify the effect of PACA on the expression of M1 biomarker CD80 and pro-resolving macrophage biomarker CD163. As shown in Figure 3B, PACA reduced the number of CD80^+^ M1 macrophages from 75.8% to 69.4% against the stimulation with LPS and IFN-γ, while increased the number of CD163^+^ macrophages from 10.5% to 24.5%. Thirdly, we further determined the effects of PACA on other macrophage biomarkers by qRT-PCR technique. As shown in Figure 3C, PACA effectively suppressed the mRNA expression of M1 biomarkers including IL-1β, CCL2, IL-6, TNF-α, CXCL10 and iNOS induced by LPS in RAW264.7 macrophages. In contrast, PACA profoundly upregulated the mRNA expression of pro-resolving M2a biomarkers (e.g., FZZ1, YM-1, IL-10 and Arg1).

### PACA decreased the expression levels of pro-fibrotic factors

**Figure 4:**
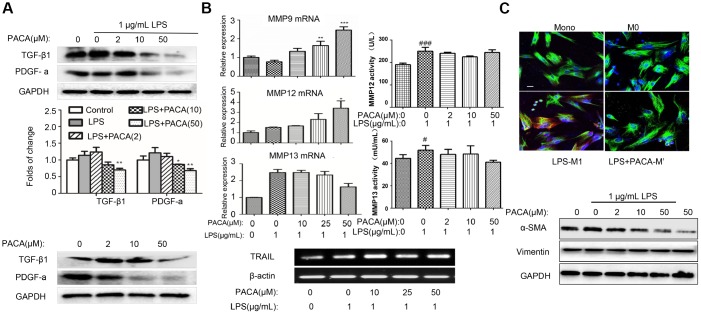
**Inhibitory effects of PACA on the expression of pro-fibrotic factors and the activation of cardiac fibroblasts.** (**A**) Inhibitory effects of PACA on TGF-β1 and PDGF-a expression in macrophages. After the treatment with PACA ± LPS, the cellular proteins were analyzed by Western blotting with specific antibodies. Data were expressed as mean ± SD (n = 3), *, p<0.05; **, p<0.01 (PACA+LPS vs LPS alone). (**B**) qRT-PCR analysis of the mRNAs for MMP9, MMP12, MMP13 and TRAIL. After the treatment with PACA and LPS, the RNAs were isolated and analyzed by qRT-PCR technique. MMP12 and MMP13 activities in the culture medium were measured by ELISA kit. Data were expressed as mean ± SD (n = 3 or n = 4). *, p<0.05; **, p<0.01; ***, p<0.001 (PACA+LPS vs LPS alone); #, p<0.05, ###, p<0.001 (Ctrl vs LPS alone). (**C**) Disruption of macrophage-mediated signals against the activation and survival of cardiac fibroblasts. The conditioned medium was prepared by treating RAW264.7 macrophages with PACA ± LPS. Adult rat cardiac fibroblasts were treated with the conditioned macrophage medium. Cardiac fibroblasts were subsequently stained with antibodies against vimentin and α-SMA and followed by the detection with fluorophore-labelled secondary antibodies. The images were captured under a fluorescence microscope. Green, vimentin; Red, α-SMA; Blue, DAPI. Scale bar: 20 μm. α-SMA expression was also analyzed by Western blotting with specific antibody.

To examine the effects of PACA on cardiac fibrosis, we determined the expression of pro-fibrotic factors including TGF-β1 and PDGF-a in different phenotypes of macrophages. As shown in Figure 4A, PACA markedly reduced the expression of TGF-β1 and PDGF-a both in control cells and LPS-challenged cells in a concentration-dependent manner. Furthermore, we examined the effects of PACA on the factors (e.g., MMP-12 and MMP-13) for tissue remodeling. As shown in Figure 4B, PACA could maintain or slightly increase the expression levels of MMP-12 and MMP-13 in LPS-induced macrophages. And the results of enzyme activities showed that PACA could maintain the activities of LPS-induced MMP12 and MMP 13 in the culture medium. Interestingly, PACA upregulated the expression of MMP-9 and TRAIL in LPS-stimulated macrophages.

### Cardiac fibroblasts were inactivated in the conditioned medium from macrophages following co-stimulation with PACA and LPS

To explore the interactions between macrophages and fibroblasts, we examined whether macrophages could regulate the activities of fibroblasts. Fibroblasts were cultured in the conditioned medium generated from LPS-stimulated macrophages. Biomarker α-SMA was detected as the index of fibroblast activation whereas vimentin served as an indicator for fibroblasts. As shown in Figure 4C, although vimentin expression was not altered, fibroblasts grown in the conditioned medium from LPS-stimulated macrophages markedly increased α-SMA expression. Interestingly, fibroblasts grown in the conditioned medium from PACA plus LPS co-stimulated macrophages showed much less increase in α-SMA expression. Moreover, we verify the expression of α-SMA in fibroblasts by Western blotting. As result, the levels of α-SMA expression appeared to be inversely correlated with the concentrations of PACA in the treatment of LPS-stimulated macrophages. These results indicated that PACA could work together with various factors secreted from M2a macrophages to control the activation of cardiac fibroblasts upon LPS challenge.

### Identification of PPAR-γ as predominant PACA-modified protein

**Figure 5:**
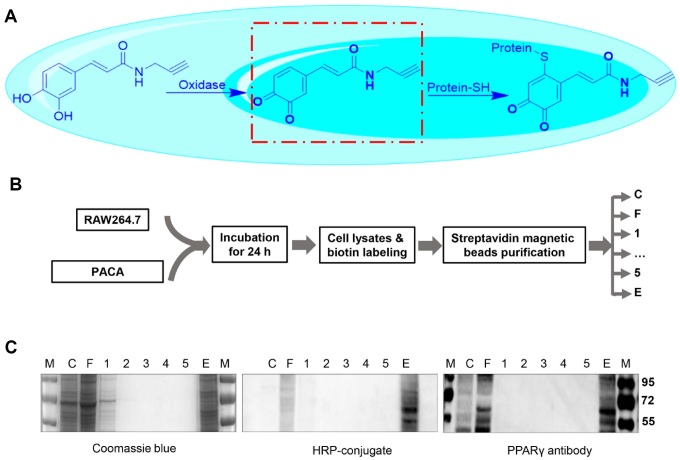
**Identification of PACA-modified proteins.** (**A**) Scheme illustrating the oxidation of PACA for reacting with cysteine residue in the proteins. (**B**) Experimental procedure for the identification of PACA-modified proteins. RAW264.7 macrophages were treated with 50 μM PACA for 24 h. The cellular proteins were biotinylated with azido-biotin under chemistry conditions and separated by streptavidin-coated magnetic beads. (**C**) Detection of PACA-modified proteins. The protein fractions were resolved by SDS-PAGE and stained with Coomassie Brilliant blue R-250, by streptavidin-HRP conjugate or by antibody against PPAR-γ. M, protein marker; C, cell lysates; F, flow-through, W1-W5, washing fraction 1 to 5; E, elution.

To explore whether PACA could form covalent conjugate with signaling proteins, biotin tag was introduced to PACA-modified proteins via standard Click chemistry method as previously described [23]. In practical, the cellular proteins were extracted from PACA-treated RAW264.7 macrophages and subsequently incubated with azido-biotin for Click chemistry biotinylation. Biotinylated proteins were isolated by streptavidin-coated magnetic beads, resolved by 10% SDS-PAGE and transferred onto PVDF membranes. As shown in Figure 5A and 5B, Western blotting with HRP−streptavidin conjugate showed a predominant protein band with the size of ~57 kDa. As shown in Figure 5C, the protein band was also detected by PPAR-γ antibody.

### PACA regulated macrophage phenotypic switch by activation of PPAR-γ signaling pathway

**Figure 6:**
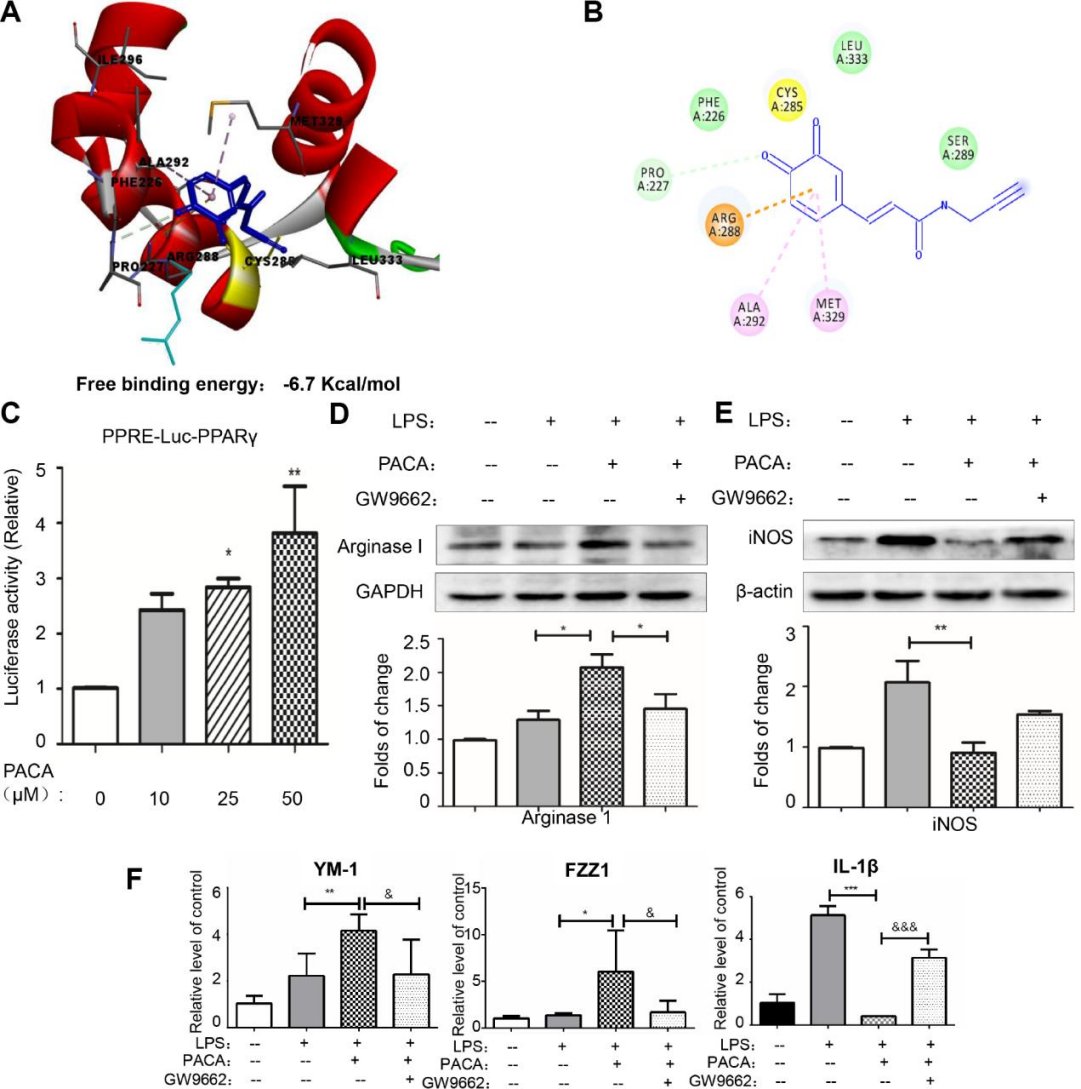
**PACA as a covalent activator for PPAR-γ.** (**A**) Docking of OXO-PACA to PPAR-γ LBD. OXO-PACA was docked to PPAR-γ LBD (PDB:) by Autodock vina. (**B**) Amino acid residues for recognizing OXO-PACA. Hydrogen bonds are shown in green, whereas pi-alkyl interactions are shown in pink. (**C**) Luciferase assay for PPAR-γ activation. RAW264.7 macrophages were transiently transfected with PPRE-X3-TK luciferase and pRL control using Effectene transfection reagent from Qiagen. Transfected macrophages were treated with PACA for 24 h. Luciferase activities were measured using the Dual-Luciferase reporter assay system. Data were expressed as mean ± SD (n = 5). * p<0.05, **, p<0.01 (PACA+LPS vs LPS alone). (**D**–**F**) PPAR-γ dependence in PACA-regulated expression of macrophages biomarkers. RAW264.7 cells were pretreated with 10 μM GW9662 for 1 h, treated with 50 μM PACA for 2 h, and stimulated with 1 μg/mL LPS for another 24 h. iNOS and Arg1 were determined by Western blotting analysis. The blots were quantified by a densitometric method. The mRNAs for M2a biomarkers (Ym-1 and FZZ1) and M1 biomarker (IL-1β) were determined by qRT-PCR. n = 3, * p<0.05, **, p<0.01, ***, p<0.01 (PACA+LPS vs LPS alone). &, p<0.05, &&, p<0.01, &&&, p<0.001 (PACA+LPS vs LPS +PACA+GW9662).

To simulate the binding of PACA to PPAR-γ, PACA and oxidized PACA (OXO-PACA) were docked into the binding site of PPAR-γ. As shown in Figure 6A and 6B, OXO-PACA exhibited high affinity towards PPAR-γ with the free binding energy of -6.7 Kcal/mol. Specifically, OXO-PACE formed 1 conventional hydrogen bond with PRO-227, 1 pi-cation interaction with ARG288 and 2 pi-alkyl interactions with MET-329, LEU-333 and ALA-292. Interestingly, CYS-285 was nearby the functional group of o-quinones, and existed in the binding pocket in PPAR-γ LBD.

To define the biological importance of covalent PACA-PPAR-γ conjugation, we assayed the promoter activity of PPAR-γ with PPRE X3-TK-luciferase expression system. As shown in Figure 6C–6E, PACA could enhance PPAR-γ luciferase activity in a concentration-dependent manner. PPAR-γ specific antagonist GW9662 effectively suppressed PACA-induced activation of PPAR-γ. Moreover, GW9662 also blocked the effects of PACA on the upregulation of Arg1, YM-1 and FZZ1 in LPS-stimulated macrophages and stopped the reduction of iNOS and IL-1β induced by PACA.

## DISCUSSION

Macrophage polarization is linked to the inflammatory response, oxidative injury and fibrosis in acute myocardial infarction [4, 25, 26]. Pharmacological approaches are pressingly needed to induce the phenotypic switch of cardiac macrophages from pro-inflammatory M1 to pro-resolving macrophage type in a timely fashion [4]. We recently synthesized and evaluated two novel caffeic acid derivatives PACA and PCA for antioxidant, anti-inflammatory and cytoprotective activities [22–24]. The long-term goal of the present study was to examine whether PACA could help the resolution of inflammation and the suppression of cardiac fibrosis. We initially discovered the potential of PACA against isoproterenol-induced cardiac fibrosis in rats. We further examined the *in vivo* and *in vitro* effects of PACA on macrophage polarization and the underlying mechanisms. The key finding from the present study was that PACA might skew macrophage polarization towards a pro-resolving macrophage phenotype via covalent binding to PPAR-γ.

Cardiac fibroblasts are overactivated in myocardial infarction or other heart diseases [27]. In response to the pathological stimuli, activated fibroblasts proliferate and produce extracellular matrix, which drives the formation of scars and ultimately leads to cardiac fibrosis [28, 29]. The drug ISO is well-known to induce cardiac fibrosis and thereby widely used to induce myocardial infarction in rodent model [30, 31]. The present study employed multiple approaches including H&E staining, Masson’s trichrome staining, immunohistochemical staining and Western blot analysis to study the effects of PACA on cardiac fibrosis in a rat model of ISO-induced myocardial infarction. As result, the results of pathological examination and α-SMA detection confirmed the successful induction of macrophage infiltration and cardiac fibrosis by ISO. PACA not only reduced α-SMA expression and cardiac fibrosis against ISO toxicity in rats, but also suppressed the activation of cardiac fibroblast and skewed macrophage polarization towards a pro-resolving macrophage phenotype. On the other hand, TGF-β1 and PDGF are known to promote the production and deposition of extracellular matrix elements such as collagen and initiate fibrosis in several organs including liver, lung, skin and heart [32, 33]. It was recently demonstrated that PDGF-a and PDGF-b could induce cardiac hypertrophy and fibrosis in transgenic mice [34]. In the present study, PACA could reduce the expression of TGF-β1 and PDGF-a in macrophages. Thus, PACA might suppress cardiac fibrosis via skewing macrophage polarization towards a pro-resolving macrophage phenotype with new low expression of TGF-β1 and PDGF-a.

Phenotypic plasticity of macrophages is tightly regulated by the contents of the pathological environment for either initiating or resolving the inflammatory responses. The overactivation of M1 macrophage polarization causes tissue damages whereas the delay of macrophage polarization towards a pro-resolving macrophage phenotype may impair tissue repair [35]. In the present study, we examined whether PACA could suppress M1 macrophage polarization and promote pro-resolving macrophage polarization. Immunohistochemical staining of macrophage biomarkers revealed that PACA effectively reduced the number of ED1^+^ cells and increased the number of ED2^+^ cells in the rat model of myocardial infarction. Consistently, upon the *in vitro* treatment of LPS-stimulated macrophages, PACA inhibited the induction of M1 macrophage biomarkers including CCL2, CXCL10, IL-1β, IL-6, iNOS and TNF-α, while enhanced the expression of M2a macrophage biomarkers including Arg1, FZZ1, YM-1 and IL-10. It was recently demonstrated that macrophages overexpressing Arg1 attenuated helper T cell2 (Th2)-dependent inflammation and fibrosis [36, 37]. Thus, resolving M2a macrophages may excise the anti-inflammatory and anti-fibrotic powder through cross-talking with other cardiac cell types. Indeed, PACA markedly increased the contents of Arg1 expression and reduced the contents of iNOS expression in cardiac macrophages after ISO injury. Beyond the capacity as one of the key M2a macrophage biomarkers, Arg1 was found to play an important role in cardiovascular diseases, especially in healing of post-infarction hearts [37]. Unexpectedly, our effort to determine Arg1 activity in heart tissues did not reveal changes between the groups with/without PACA treatment. Collectively, these results suggested that PACA might elicit anti-inflammatory and anti-fibrotic activities through regulating macrophage polarization, highlighting the potential of PACA in the treatment of cardiac fibrosis in myocardial infarction.

**Figure 7:**
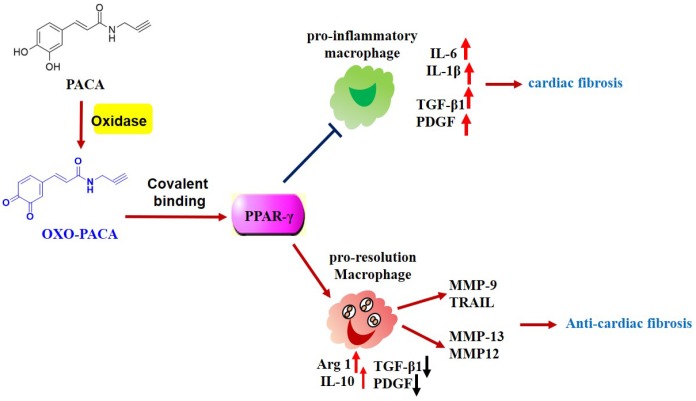
**Potential mechanisms underlying the anti-inflammatory and anti-fibrotic activities of PACA.** PACA is oxidized to o-quinone, forms covalent conjugate with PPAR-γ and consequently activates PPAR-γ pathway. PPAR-γ activation promotes pro-resolving macrophage polarization, suppresses the expression of TGF-β1, PDGF-a, and maintains the expression of MMPs. Ultimately, PACA attenuates the activation of cardiac fibroblast and the progression of cardiac fibrosis.

PPAR-γ is one of the most important signaling pathways for controlling macrophages polarization [38, 39]. The activation of PPAR-γ is known to promote anti-inflammatory M2 macrophage polarization based on the previous studies in atherosclerosis [15, 16]. In the present study, we identified PPAR-γ as a major cellular protein that was covalently modified by PACA. Following 24 h treatment with PACA, macrophages were lysed and biotinylated with biotin azide via a copper-catalyzed click reaction. As result, PACA-modified proteins were efficiently biotinylated for affinity enrichment with streptavidin-coated magnetic beads. A predominant band of ~57 kDa was detected by Western blotting with HRP−streptavidin conjugate and PPAR-γ specific antibody. Based on molecular docking results, OXO-PACA has higher affinity for binding to PPAR-γ and cysteine residue Cys285 resides within the binding site in PPAR-γ LBD. Nevertheless, previous studies demonstrated that endogenous unsaturated fatty acids and other agonists could covalently bind to Cys285 and subsequently enhanced the transcriptional activity of PPAR-γ [40, 41]. It is not surprising that the intracellular oxidases oxidize catechol-bearing natural products into o-quinones, rendering the stronger reactivity to the cysteine residues in various proteins [42]. Thus, we deduced that OXO-PACA could activate PPAR-γ via covalent modification of Cys285. Indeed, the luciferase reporter assay demonstrated that PACA could induce PPAR-γ activation. Moreover, PPAR-γ specific antagonist GW9662 not only disrupted the effects of PACA on LPS-induced iNOS expression and IL-1β mRNA expression but also abolished the activity of PACA to upregulate Arg1 expression, Ym-1 and FZZ1 mRNA expression. Thus, these results suggested that PACA suppressed M1 macrophage polarization and enhanced pro-resolving M2a macrophage polarization in a PPAR-γ dependent manner (Figure 7).

In conclusion, the present study demonstrated that PACA effectively attenuated cardiac fibrosis and skewed macrophage polarization towards a resolving M2a-like phenotype via covalent binding to PPAR-γ. Ultimately, these results may pave the avenue to develop PACA as a new therapeutic reagent for the treatment of cardiac fibrosis and related cardiovascular diseases.

## MATERIALS AND METHODS

### Chemicals and reagents

PACA was synthesized and characterized by NMR and MS as previously described [22, 23]. Cell culture medium, fetal bovine serum (FBS) and penicillin/streptomycin were purchased from Invitrogen (Carlsbad, CA, USA). Antibodies against iNOS, cyclooxygenase-2 (COX-2) and glyceraldehydes-3-phosphate dehydrogenase (GAPDH), and PPAR-γ was purchased from Cell Signaling Technology (Boston, MA, USA). The antibodies against Arg1, ED2 and CD163 were obtained from Santa Cruz Biotechnology (Santa Cruz, CA, USA). Antibodies against ED1 and CD68 were purchased from Abcam (Cambridge, Massachusetts, UK). Anti-mouse CD80 FITC was obtained from BD Biosciences (San Diego, CA, USA). Alexa Fluor 594-conjugated goat anti-rabbit IgG antibody and Alexa Fluor 488-conjugated goat anti-mouse IgG secondary antibody were purchased from Life Technologies (Carlsbad, CA, USA). Anti-rabbit HRP-conjugated IgG secondary antibody and other chemicals including GW9662, a specific PPAR-γ inhibitor, were obtained from Sigma-Aldrich (St. Louis, MO, USA).

### Cell culture and drug treatment

Murine macrophage cell line RAW264.7 was obtained from ATCC (Manassas, VA, USA) and maintained in Dulbecco's Modified Eagle Medium (DMEM) containing 10% FBS and 1% penicillin/streptomycin at 37 °C in a humidified 5% CO2 atmosphere. For drug treatment, the cells were grown to 70-80% confluence in the complete growth medium and treated with PACA at the indicated concentrations for the specified times, whereas the control cells were treated with equal amount of dimethyl sulfoxide (DMSO) under the same conditions. For Western blot analysis, the cells were pre-treated with PACA for 2 h and subsequently stimulated with LPS for another 24 h.

For preparation of macrophage-conditional medium, RAW264.7 macrophages were pretreated with PACA for 2 h, and then stimulated with LPS for another 24 h. After incubation, the culture medium was collected as conditioned medium. And the conditioned medium was added into cardiac fibroblasts in order.

### Real-time quantitative RT-PCR (qRT-PCR) analysis

Following the treatment with PACA, the total RNAs were isolated with TRIzol reagent from Invitrogen (Carlsbad, CA, USA) following the manufacture's instruction, and converted into cDNA using RevertAid RT kit from Thermo Scientific (Waltham, MA, USA) as previously described [43]. The mRNAs of IL-6, iNOS, IL-1β, TNF-α, CCL2, CXCL10, IL-10, Arg1, MMP-9, MMP-12, MMP-13 and GAPDH were analyzed by qRT-PCR technology with gene specific QuantiTect Primer^®^ and QuantiTect SYBR Green PCR Kit from Qiagen (Hilden, Germany). Target mRNA levels were normalized to the geometric mean of GAPDH mRNA levels.

### Western blot analysis

Following drug treatment, the cellular proteins were extracted and analyzed by Western blotting technique for the expression levels of specific proteins as previously described [44, 45]. Briefly, the cellular proteins were extracted and resolved by electrophoresis on 10% SDS-polyacrylamide gels and transferred onto a polyvinylidenedifluoride (PVDF) membrane. After overnight incubation in 5% non-fatted milk powder in the mixture of Tris-buffered saline and Tween-20 (TBS-T), the membranes were probed with specific primary antibodies, and subsequently detected by goat anti-rabbit or goat anti-mouse IgG-HRP conjugate secondary antibodies. The activity of peroxidase on the blots was visualized with enhanced chemiluminescence (ECL) detection reagents from GE Healthcare (Uppsala, Sweden) according to the manufacturer’s instruction.

### Cell immunofluorescence staining

Following drug treatment, the cells were fixed with 4% paraformaldehyde in PBS for 30 minutes, permeabilized with 0.5% Triton X-100 for 10 minutes and blocked in 5% normal goat serum in PBS for 1 h at room temperature. The cells were probed with anti-α-SMA and anti-vimentin antibodies, and subsequently detected by Alexa Fluor 594-conjugated goat anti-rabbit or Alexa Fluor 488-conjugated goat anti-mouse IgG secondary antibody. After staining the cell nuclei with DAPI, the immunofluorescence were imaged under Carl Zeiss LSM microscopy (Jena, Germany).

### Flow cytometry

RAW 264.7 macrophages were pretreated with 50 μM PACA for 2 h and followed by LPS (1 μg/mL) and IFN-γ (0.5 μg/mL) stimulation for another 24 h. After incubation, the cells were collected and stained with fluorophore-conjugated antibody against biomarker CD80. As for biomarker CD163 staining, the cells were incubated with primary antibody against CD163. After three washes, the cells were incubated with fluorophore-conjugated secondary antibody. Flow cytometric analysis was carried out on a BD LSR Fortessa Analyzer from BD Biosciences (San Jose, CA, USA) and the data were analyzed with FlowJo software (Tree Star, Ashland, OR).

### Luciferase reporter assays

RAW264.7 macrophages in 12-well plated were co-transfected with 300 ng of PPRE-X3-TK controlled Firefly luciferase plasmid from Addgene (Cambridge, MA, USA) and 30 ng of pRL-TK-renilla luciferase plasmid using Effectene transfection reagent from Qiagen (Hilden, Germany). After 24 h cell culture, the cells were treated with PACA at different concentrations for another 24 h. The activities of luciferase were detected by using a Dual-luciferase reporter assay system from Promega (Madison, WI, USA) with a 96-well microplate Luminometer according to the manufacturers’ instructions. The activity of Firefly luciferase was normalized to that of Renilla luciferase to control for sample-to-sample variations in transfection efficiency.

### Isolation of cardiac fibroblasts from adult rats

Cardiac fibroblasts were isolated from adult rata and cultured as previously described [46]. Briefly, male Sprague-Dawley rats (8–10 weeks old) were anesthetized with ketamine (90 mg/kg, i.p.) and xylazine (10 mg/kg, i.p.). The hearts were quickly collected and placed in cold Krebs Henseleit Buffer (KHB). The atria and valves were removed. The ventricles were minced into small pieces in sterile KHB, and then digested with 0.1% collagenase/KHB for 15 min at 37 °C. The digestion was repeated for 6 times until the complete digestion. Following the centrifugation at 400 × *g* for 10 minutes, the cell pellet was suspended in DMEM with 5 mM glucose, 10% FBS, 100 U/ml penicillin, 100 μg/ml streptomycin and 100 μg/ml ascorbic acid. After 4 h incubation at 37°C in cell culture cabinet, the unattached cells were removed. The attached cells were cultured while the medium was changed three times a week. The first passage was made when cell culture reached 80% confluence. After another 5-day cell culture, the second passage was made. The cardiac fibroblasts were identified by positive staining for the fibroblast biomarker vimentin.

### Animal models of isoproterenol-induced myocardial infarction

The procedures for animal experiments were approved by the Committee on the Use of Live Animals in Teaching and Research of the University of Hong Kong (CULATR 3803-15). Prior to animal experiments, isoproterenol was dissolved in 0.9% saline while PACA was dissolved in 0.9% saline in containing 0.5% ethanol. For the animal experiments, male Sprague–Dawley rats were randomly assigned to the following five groups: control group, receiving intraperitoneal (IP) injection of 0.9% saline and vehicle (0.5 ml/day) for 7 days; isoproterenol group, receiving subcutaneous (SC) injection of isoproterenol (100 mg/kg/day) for 2 consecutive days from the 1^st^ day; isoproterenol + PACA (10, 20 and 40 mg/kg/day) groups, receiving oral administration of PACA for 7 consecutive days (at 10 minutes prior to isoproterenol treatment). The rats were sacrificed on day 0, day 1, day 3, day 5, day 7, day 14, day 21 and day 35.

### Histopathological examination

Histopathological examination was performed as previously described [47]. At the end of drug treatment, animals were euthanized. Cardiac samples were collected from four groups, fixed in 4% paraformaldehyde in PBS and embedded in paraffin. Paraffin-embedded sections (5-μm) were prepared, stained with hematoxylin and eosin (H&E) stain and imaged under light microscope. The images were assessed for gross myocyte injury and the effects of interventions.

### Immunohistochemistry staining

Paraffin-embedded sections (5-μm) were dewaxed, rehydrated, and subjected to antigen retrieval in 10 mM citrate buffer (pH 6.0) in a microwave oven. The tissue specimens were incubated in 0.3% H_2_O_2_ for 10 minutes, washed with PBS for three times and blocked with 5% goat serum in TBS-T buffer overnight. Macrophage biomarkers (i.e., ED1 or ED2) were probed with primary antibodies and detected by biotin-labeled secondary antibody. Biotin retention was visualized by HRP-streptavidin conjugate and ImmunoCruz mouse ABC Staining System (sc-2017) from Santa Cruz. The slides were imaged on a Zeiss fluorescence microscopy from Carl-Zeiss (Jena, Germany).

### Isolation and identification of covalent PACA–protein conjugates

RAW264.7 macrophages (2 × 10^7^) were treated with 50 μ M PACA for 24 h and subsequently lysed in 0.6 mL of RIPA buffer containing protein inhibitor and 100 mM iodoacetate amide, then centrifugated at 13000 rpm for 15 min. After desalting with 10 kDa Microcon® centrifugal filter, the protein mixture was incubated with 100 μ M azido-biotin in a Click chemistry reaction mix including 1 mM sodium ascorbate, 100 μ M tris [(1-benzyl-1H-1,2,3-triazol-4-yl)methyl]amine, 1 mM CuSO_4_ at RT for 4 h. Following desalting with 10 kDa centrifugal filter, the reaction mixture was incubated with 50 μL streptavidin-coated magnetic beads from Thermo Fisher Scientific (Waltham, MA, USA) at RT for 1 h and at 4 °C for 3 h. The cellular proteins were separated into flow-through (FL), wash fractions 1-5 (W1-5) and elution (E). The resulted fractions were resolved in a 10% SDS-PAGE gel, stained with Coomassie Brilliant blue R-250 from Bio-Rad Laboratories (Hercules, CA, USA), and detected with HRP-streptavidin conjugate. For confirmation, the blot was probed with anti-PPAR-γ primary antibody and detected by HRP-conjugated secondary antibody. The blots were visualized with enhanced chemiluminescence (ECL) detection reagents from GE Healthcare (Uppsala, Sweden).

### Molecular docking

The crystal structure (PDB: 2q5s) of PPAR-γ ligand-binding domain (LBD) was downloaded from RCSB PDB website (http://www.rcsb.org/pdb). The chemical structure of the ligand molecule OXO-PACA was generated by ChemBioDraw Ultra 12.0 software. The protein-ligand interactions were simulated by AutoDock Vina in PyRx-virtual screen tool package [48]. Prior to docking all water molecules were removed from PPAR-γ LBD structure. OXO-PACA was introduced to grid box corresponding to the binding site of PPAR-γ LBD. The docking results were analyzed by Discovery Studio Visualizer software.

### Measurement of MMP enzyme activities

After drug treatment, the culture medium was collected for MMP enzyme activity assay. MMP12 and MMP13 enzyme activities were determined by mouse ELISA kit (Share Ltd., Shanghai, China).

### Statistical analysis

Results represent the means ± SD or mean ± SEM from at least three independent experiments. Data was analyzed by one-way analysis of variance (ANOVA), followed by Dunnett's test or LSD's test with Graphpad Prism 5 (GraphPad Software, Inc, La Jolla, CA, USA). A p-value of < 0.05 was considered as statistical significance.
